# Delta chirality ruthenium ‘light-switch’ complexes can bind in the minor groove of DNA with five different binding modes

**DOI:** 10.1093/nar/gkw753

**Published:** 2016-09-05

**Authors:** James P. Hall, Páraic M. Keane, Hanna Beer, Katrin Buchner, Graeme Winter, Thomas L. Sorensen, David J. Cardin, John A. Brazier, Christine J. Cardin

**Affiliations:** 1 Department of Chemistry, University of Reading, Whiteknights, Reading, RG6 6AD, UK; 2 Diamond Light Source, Harwell Science and Innovation Campus, Fermi Avenue, Didcot, OX11 0DE, UK; 3 Department of Pharmacy, University of Reading, Whiteknights, Reading, RG6 6AD, UK

## Abstract

[Ru(phen)_2_(dppz)]^2+^ has been studied since the 1990s due to its ‘light-switch’ properties. It can be used as a luminescent DNA probe, with emission switched on through DNA binding. The luminescence observed is dependent on the solvent accessibility of the pyrazine nitrogen atoms, and therefore is sensitive to changes in both binding site of the cation and chromophore orientation. The compound is also chiral, and there are distinct differences between the enantiomers in terms of the emission behaviour when bound to a variety of DNA sequences. Whilst a number of binary DNA-complex X-ray crystal structures are available, most include the Λ enantiomer and there is very little structural information about binding of the Δ enantiomer. Here, we present the first X-ray crystal structure of a Δ enantiomer bound to well-matched DNA, in the absence of the other, Λ enantiomer. We show how the binding site observed here can be related to a more general pattern of motifs in the crystallographic literature and propose that the Δ enantiomer can bind with five different binding modes, offering a new hypothesis for the interpretation of solution data.

## INTRODUCTION

**Figure 1.**
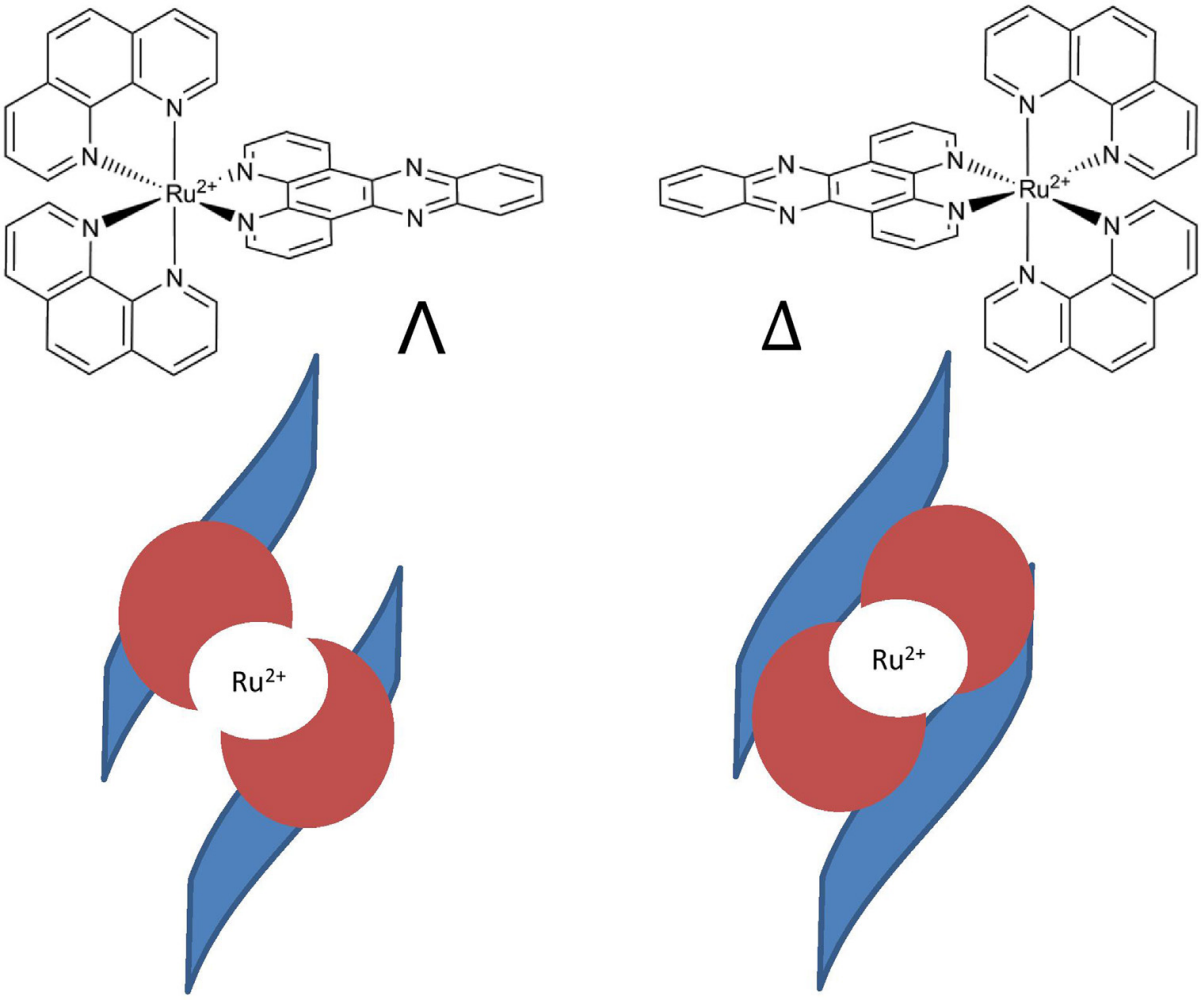
(Left) Λ and (Right) Δ-[Ru(phen)_2_(dppz)]^2+^. It has been previously proposed that the Λ enantiomer will intercalate less well into DNA than the Δ, as the twist of the complex does not complement the helix sense of the DNA (6). In the schematic diagram, phen groups are represented by red circles with the DNA backbone drawn as blue ribbons.

The binding of ligands to DNA can have a profound effect on the resulting DNA structure, depending on the binding mode. Attachment modes can include groove binding, where the molecule sits in either of the two grooves, and intercalation, where a cationic planar ligand lies between neighbouring DNA base-pairs. Ruthenium–polypyridyl complexes are one class of DNA intercalators that have been studied intensely since the mid-1980s (1,2). Octahedral Ru-dppz complexes are of particular interest, as they combine the ability to intercalate with a photophysical ‘payload’, namely the ability to act either as a luminescent DNA probe (3) or to interact directly with DNA via photooxidation of guanine bases (4). One complication with studying such complexes in the presence of DNA is that they are chiral molecules (Figure 1), which are stable as resolved enantiomers, with each bound enantiomer displaying different photophysical properties as a consequence of different binding modes (5).

One such ruthenium polypyridyl is [Ru(phen)_2_(dppz)].Cl_2_. This compound is non-luminescent in water but is able to luminesce in the presence of DNA and is therefore referred to as a DNA light-switch compound. Whilst several X-ray crystal structures containing the Λ enantiomer are available (7,8), there are no published atomic coordinates of a crystal structure showing how the Δ enantiomer on its own can bind to well matched DNA, in the absence of an adjacent Λ complex. This is despite a great deal of discussion in the literature, due to differences in the luminescence lifetimes of the bright state observed in solution, about how the enantiomers of [Ru(phen)_2_(dppz)]^2+^ and the closely related [Ru(bpy)_2_(dppz)]^2+^ can bind to DNA (9,10). The Δ enantiomer typically shows a stronger luminescence and, perhaps related to this observation, it has been previously hypothesised that the binding of a Λ enantiomer of an octahedral ruthenium polypyridyl complex would be less favourable than the Δ. The assumption has been that the twist of the Δ enantiomer is a better fit to the right-handed helix sense of the DNA backbone (6) and could therefore intercalate more effectively. Since then, the binding modes of intercalation and semi-intercalation have been predicted (11) and observed by X-ray crystallography (12). Unexpectedly, the Λ-enantiomer was often found to crystallise selectively with particular DNA sequences when starting with racemic mixtures of enantiomers, a contradictory result possibly due to a more favourable crystal packing with the Λ. It was therefore of great interest to establish why the Δ enantiomer was more difficult to crystallise, even when starting from a pure enantiomer in the crystallisation trials. It is important to understand how the complexes bind to DNA, as we have found these insights to be key for the interpretation of results from spectroscopic binding and ultrafast kinetic studies (13).

The binding of Δ-[Ru(bpy)_2_(dppz)]^2+^ inserted into DNA containing multiple AA mismatch sites was recently described, in which the complex bound through metalloinsertion with simultaneous π-stacking between the bpy groups and ‘flipped out’ adenine bases (14). In contrast to this, the interaction of the Λ enantiomer appears relatively straightforward: Λ-[Ru(phen)_2_(dppz)]^2+^ has been shown to intercalate into well-matched DNA and, both it and the isostructural Λ-[Ru(TAP)_2_(dppz)]^2+^, bind selectively to 5′-TA-3′ steps and not 5′-AT-3′ in both the crystal state and solution (15). The effects of introducing methyl (16) and chloro (17) substitutions onto the dppz group have also been examined, and the resulting DNA structures were isomorphous with those reported for the parent compound, while displaying site preferences for asymmetric substitution.

Here, we report two crystal structures of Δ-[Ru(phen)_2_(dppz)]^2+^ in the presence of an oligonucleotide decamer that may help with interpreting the photophysics of the compound when bound to DNA in solution.

## MATERIALS AND METHODS

Δ-[Ru(phen)_2_(dppz)].Cl_2_ was produced and purified using the method previously reported (8). Crystals yielding both structures 1 (DNA + Δ-[Ru(phen)_2_(dppz)]^2+^ + Ba^2+^) and 2 (DNA + Δ-[Ru(phen)_2_(dppz)]^2+^ + [Co(NH_3_)_6_]^3+^) were obtained in a similar fashion. Both were crystallised using sitting-drop vapour diffusion. Crystals giving structure 1 were grown by mixing 1 μl 2 mM d(TCGGCGCCGA), 1 μl 4 mM Δ-[Ru(phen)_2_(dppz)].2Cl and 6 μl of a solution containing 20 mM BaCl_2_, 12 mM spermine-tetra HCl, 40 mM sodium cacodylate pH 7, 80 mM KCl and 10% (v/v) 2-methyl-2,4-pentanediol. This was equilibrated against 500 μl of 35% 2-methyl-2,4-pentanediol at 18°C for 2 weeks. Crystals yielding structure 2 were grown by mixing 1 μl 2 mM d(TCGGCGCCGA), 1 μl 2 mM Δ-[Ru(phen)_2_(dppz)].Cl_2_ and 6 μl of a solution containing 40 mM sodium cacodylate pH 5.5, 20 mM cobalt hexammine, 12 mM NaCl, 80 mM KCl and 10% 2-methyl-2,4-pentanediol. This was equilibrated against 500 μl of 35% 2-methyl-2,4-pentanediol.

### Structure solution for d(TCGGCGCCGA)_2_ with Δ-[Ru(phen)_2_(dppz)]^2+^ in the presence of BaCl_2_

The data were collected on beamline I02 at Diamond Light Source using radiation with a wavelength of 0.866 Å at 100 K; 90° of data were collected in 180 images with a 0.5 s exposure time. The data were processed using xia2 (18), with XDS (19) and XSCALE to give 20 581 unique reflections. The data had an outer shell resolution of 0.97 Å with an outer shell I/σI of 1.9 and multiplicity of 6.2.

Initial phases were found, using the anomalous scattering of barium, with SHELXC/D/E (20) via CCP4i (21). The structure was built using Coot (22) and refined using phenix.refine (23) to give a final R_factor_ of 0.112 and R_free_ of 0.127. Full data collection and refinement statistics are in Table 1.

**Table 1.** X-ray data collection and refinement statistics

Structure	1 (With Ba^2+^)	2 (With Cobalt Hexammine)
**Data Processing**		
Space group	*P* 4_1_2_1_2	*P* 4_1_2_1_2
Resolution, Å	25.18–0.97 (1.00–0.97)	22.61–0.99 (1.02–0.99)
R_merge_	0.027 (0.857)	0.031 (0.833)
R_meas_	0.041 (0.993)	0.035 (0.964)
R_pim_	0.015 (0.368)	0.016 (0.470)
Total number of observations	132 604 (9440)	125 456 (9259)
Total number of unique observations	20 581 (1520)	19 226 (1374)
I/σI	25.3 (1.9)	19.9 (1.9)
CC_1/2_	0.999 (0.618)	1.00 (0.658)
Completeness, %	96.7 (97.3)	94.1 (92.3)
Multiplicity	6.4 (6.2)	6.5 (6.7)
Mid-slope of anom normal probability	1.39	1.18
**Refinement**		
No. Reflections	20 549	19 198
R_work_/R_free_	0.112/0.127	0.123/0.141
No. Atoms		
DNA	345^a^	474^a^
Ligands	156	161
Water	83	69
Average B-factors		
DNA	16.85	20.28
Ligands	15.05	16.03
Water	29.97	33.61
rmsd		
Bond Lengths, Å	0.013	0.013
Bond Angles, °	2.525	2.325
**PDB ID**	**5JEU**	**5JEV**

^a^Discrepancy is due to structural disorder.

### Structure solution for d(TCGGCGCCGA)_2_ with Δ-[Ru(phen)_2_(dppz)]^2+^ in the presence of [Co(NH_3_)_6_]^3+^

The data were collected on beamline I02 at Diamond Light Source using radiation with a wavelength of 0.866 Å at 100 K; 90° of data were collected in 900 images with a 0.1 s exposure time. The data collection strategy was different for this dataset than for crystals yielding structure 1 (with Ba^2+^). This discrepancy is due to the datasets being some of the first collected on I02 after the installation of a Pilatus 6M (date of data collection, 14th December, 2012) and therefore the authors were still evaluating the optimal data collection strategy. The data were processed using xia2, with XDS and XSCALE to give 19226 unique reflections. The data had an outer shell resolution of 0.99 Å with an outer shell I/σI of 1.9 and multiplicity of 6.7.

Initial phases were found, using the anomalous scattering of cobalt, with SHELXC/D/E via CCP4i. The structure was built using Coot and refined using phenix.refine to give a final R_factor_ of 0.123 and R_free_ of 0.141. Full data collection and refinement statistics are in Table 1. Coordinates and experimental data can be downloaded from www.wwpdb.org using the PDB IDs in Table 1.

## RESULTS

### Structures 1 and 2: Δ-[Ru(phen)_2_(dppz)]^2+^ bound to d(TCGGCGCCGA)_2_

**Figure 2.**
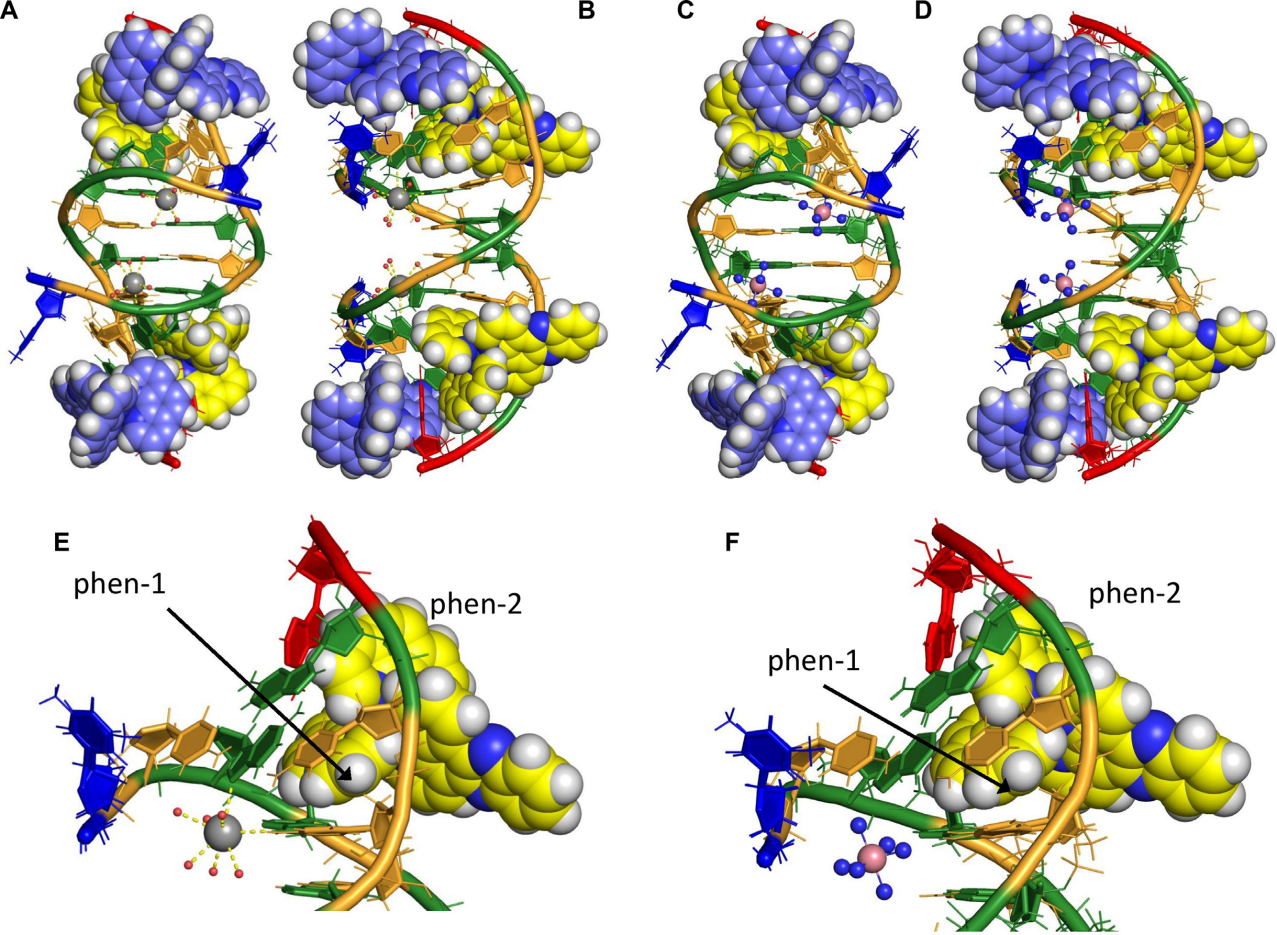
Two crystal structures showing Δ-[Ru(phen)_2_(dppz)]^2+^ bound to d(TCGGCGCCGA)_2_. (**A, B** and **E**) In structure 1, Ba^2+^ (silver sphere) is bound at the semi-intercalation sites, and has a full coordination shell of water molecules (small red spheres). (**C, D** and **F**) In structure 2, cobalt hexammine (pink spheres and small blue spheres) sits in the major groove at the semi-intercalation site. Semi-intercalation induces a 48° kink into the duplex at each binding site. Semi-intercalating complexes are coloured with carbons in yellow and end-capping complexes with carbons in blue. DNA bases are coloured according to type with G in green, C in orange, A in red and T in blue.

These are the first published crystal structures of a pure Δ enantiomer of a mononuclear ruthenium complex bound in a well-matched DNA sequence and therefore presents a unique opportunity to examine the consequences of binding by this enantiomer alone. The two structures presented here only differ, in gross structural terms, in the presence of Ba^2+^ or cobalt hexammine at the semi-intercalation site (Figure 2A–D). As such, the structures will be considered as one until the semi-intercalation subsection.

The structure shows Δ-[Ru(phen)_2_(dppz)]^2+^ bound to d(TCGGCGCCGA)_2_ by two distinctly different binding modes. The first binding mode, end capping, occurs at either end of the DNA duplex. The dppz group of the complex stacks onto both of the C_2_–G_9_ base pairs, with both the T_1_ and A_10_ flipped out. The DNA duplex therefore possesses eight base pairs with one pair of flipped-out bases at either end of the duplex. A packing diagram can be found in Supplementary Figure S1 in the Supplementary Data.

### End capping

**Figure 3.**
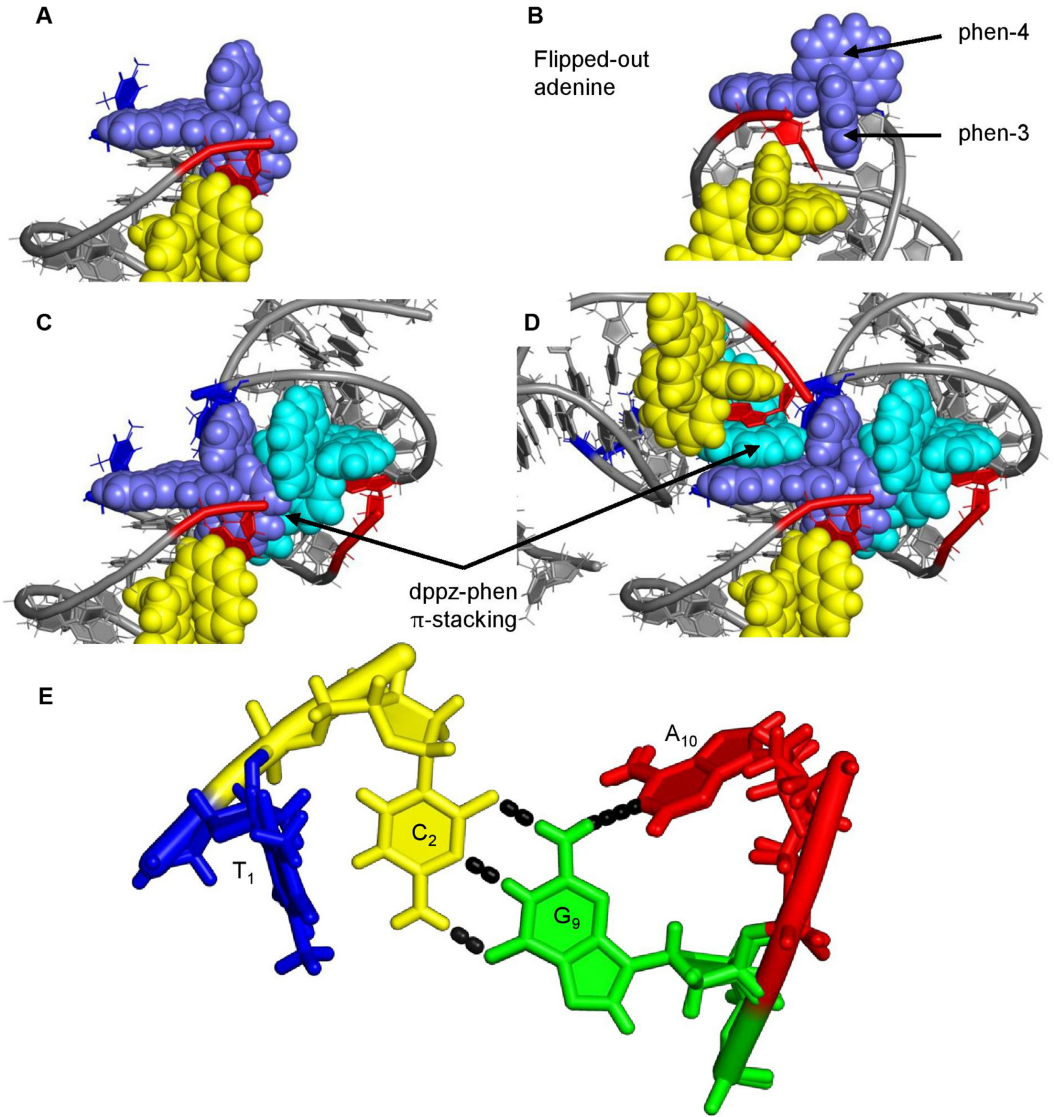
End-capping of d(TCGGCGCCGA)_2_ by Δ-[Ru(phen)_2_(dppz)]^2+^. (**A**) The dppz group from the complex (purple) stacks onto the C_2_G_9_ base pair. A_10_ (red) and T_1_ (blue) flip out. (**B**) A_10_ stacks onto one phen group from the end-capping complex and another from the semi-intercalating complex (yellow). (**C**) A dppz group from a symmetry related complex (cyan) stacks onto a phen group from the end-capping compound. (**D**) a symmetry related (4_1_ axis) phen stacks onto the dppz group of the end-stacking complex to create an assembly of stacking interactions around the end-capping site. The DNA C/G bases are drawn in grey to aid clarity (**E**) Hydrogen bonding between A_10_ and G_9_ stabilises the interaction site. A_10_ is drawn in red, T_1_ in blue, C_2_ in yellow and G_9_ in green. Hydrogen bonds are drawn as black dashed lines.

The complex binds into the minor groove and end-caps the duplex at both ends, with both T_1_ and A_10_ flipped out (Figure 3A). This has not been observed with the Λ enantiomer, although 3′-terminal adenine flipping onto a symmetry related dppz chromophore is normally observed. A_10_ π-stacks onto the DNA side of phen-3 (Figure 3B) with a dppz from a neighbouring end-stacking complex stacked on the other side (Figure 3C). T_1_ is also flipped out and stacks onto the DNA side of a symmetry related phen-4. Additionally, in place of the T_1_-A_10_ base pair, a symmetry-related phen-1 stacks onto the dppz group (Figure 3D). In the resulting assembly, the complex is end-stacked with an angle between the long axis of the dppz group and the hydrogen bond of G_2_–C_9_ of 67°, which is a more acutely angled mode than that seen for the Λ enantiomer. The directionality of the dppz is towards the side of the DNA duplex that contains the flipped-out A_10_, and similar self-stacking of the complex has been previously observed in the X-ray crystal structure of the complex in the absence of DNA (24). The position of the adenine is stabilised by a hydrogen bond between A_10_(N_1_) and G_9_(NH_2_) (Figure 3E), as well as the stacking between the base and the ancillary ligand.

### Semi-intercalation (kinking)

In both structures, the phen group (phen-1, Figure 2E and F), is inserted between the G_3_G_4_:C_7_C_8_ base pairs, in the minor groove, inducing a 48° kink in the duplex at each site. In structure 1, which contained BaCl_2_ in the crystallisation condition, Ba^2+^ coordinates to the guanine bases at the N7 position. We have previously reported this binding mode in a number of our structures with the Λ enantiomer of [Ru(phen)_2_(dppz)]^2+^ and [Ru(TAP)_2_(dppz)]^2+^ and derivatives.

However, the crystallisation conditions for structure 2 contained no BaCl_2_ but did contain cobalt hexammine. In structure 2, cobalt hexammine, with 40% occupancy, sits in the major groove (Figure 2F) and forms hydrogen bonds with bases in the floor of the groove. This does not stabilise the kink of the DNA since it is bound only through water bridges, and therefore, this is the first observation of semi-intercalation (kinking) without a base-coordinated metal cation.

The second phen group, phen-2, protrudes into the minor groove and partially π-stacks onto the flipped out A_10_. The dppz group is directed away from the DNA and stacks onto a dppz group from a symmetry equivalent complex (Supplementary Figure S2).

### DNA structural deformation

The overall structure of the DNA duplex, based on the predominant sugar pucker, is that of an A/B hybrid. This is equally applicable to both structures which are broadly consistent with each other. When a least-squared superimposition is performed, using all DNA main chain atoms, the two structures have an rmsd of 0.476 Å. However, there are slight differences between structure 1 and 2, depending on whether Ba^2+^ or [Co(NH_3_)_6_]^3+^ is present at the semi-intercalation site.

When [Co(NH_3_)_6_]^3+^ is present, the twist at the central step is 39°, 3° higher than for B-DNA. The neighbouring steps, G_4_C_5_:G_6_C_7_ and G_3_G_4_:C_7_C_8_, have twists of 28° and 23°, respectively, although the highly reduced twist at the G_3_G_4_:C_7_C_8_ step is likely to be due to the semi-intercalation interaction at this site. When Ba^2+^ is bound to G_3_ and G_4_ (structure 1), the twist at the central step is 32°. Steps G_4_C_5_:G_6_C_7_ and G_3_G_4_:C_7_C_8_ have a twist of 33° and 21°, respectively.

The Ba^2+^, acting as an anchor point, reduces the twist at the semi-intercalation site by 2°, when compared to the cobalt hexammine bound form. This seemingly small difference results in a reduced twist at the G_4_C_5_:G_6_C_7_ step which must be corrected by an increased twist at the central step. This gives both forms the same average twist over steps 3–7 of 28° but allows for a different distribution of twist angle.

At the central step the roll angle in both structures is <6° and therefore the DNA structure is not significantly kinked. This is in contrast to the small bend observed in the structure of the Λ complex with the same DNA sequence, where the roll angle is 22°. Conformational analysis was performed by W3DNA (25) and the output can be found in Supplementary Tables S1 and S2 in the Supplementary Data.

## DISCUSSION

The structures reported here show, for the first time, how an isolated Δ enantiomer of [Ru(phen)_2_(dppz)]^2+^ can bind to DNA through a non-intercalative binding mode. The complex, like the Λ enantiomer, is able to semi-intercalate into the DNA at GG steps, causing an approximately 50° kink at each step. This has also been observed with Λ-[Ru(TAP)_2_(dppz)]^2+^, a photooxidising complex, confirming that this is a generally applicable binding mode. Interestingly, the binding of the Δ enantiomer to d(TCGGCGCCGA) gives a crystal structure with the opposing screw axis to the Λ, P4_1_2_1_2 instead of P4_3_2_1_2. This is the case even though the chirality of the DNA is maintained.

**Figure 4.**
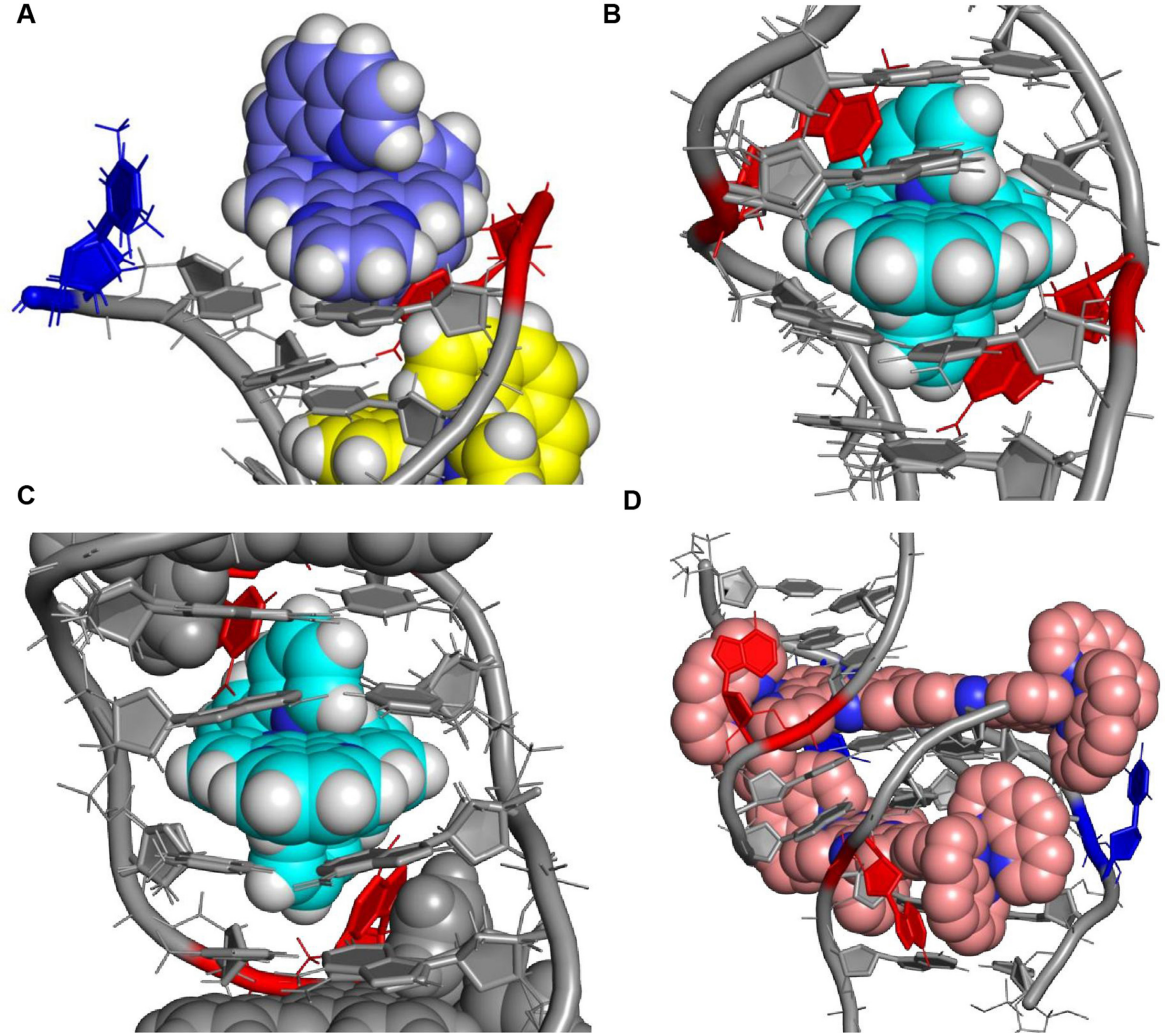
Views of four different Δ enantiomer binding sites in X-ray crystal structures. (**A**) The end-capping binding motif reported here. Adenine bases are illustrated in red with thymine in blue. As can be observed, adenine stacks onto a phen ligand whereas thymine stacks onto a symmetry related complex (not shown). Δ-[Ru(phen)_2_(dppz)]^2+^ is drawn in blue (**B**) Δ-[Ru(bpy)_2_(dppz)]^2+^ inserted into an A–A mismatch site (PDB ID 4E1U). The two mismatched adenine bases stack onto either bpy group. (**C**) Adjacent to the mismatch site, a second complex is bound by classical intercalation. The adenine bases from the mismatch site and a second mismatch site both stack onto the bpy groups of the intercalated complex, reducing intercalation depth. Δ-[Ru(bpy)_2_(dppz)]^2+^ is drawn in cyan. (**D**) Threading by Δ,Δ-[μ-(11,11′-bidppz)(phen)_4_Ru_2_]^4+^ into d(CGTACG)_2_, causing both an adenine and thymine to flip out of the DNA base stack (PDB ID 4GQJ). Both adenine and thymine stack onto the phen group in two separate complexes. Δ,Δ-[μ-(11,11′-bidppz)(phen)_4_Ru_2_]^4+^ is drawn in pink.

However, the main interest in this structure lies in the binding mode at the terminal step (Figure 4A). The stacking of symmetry-related complexes causes both T_1_ and A_10_ to flip out, with A_10_ stacking onto two phen ligands; one from the semi-intercalating complex and the other from the end-capping compound. Whilst in this structure the binding mode is technically end-capping, if this is considered to be half a picture of an intercalative binding site, then binding can be compared with existing structures.

Similar stacking, between purine bases and ancillary ligands of Δ complexes, are also observed in two other reported crystal structures – one with Δ-[Ru(bpy)_2_(dppz)]^2+^ bound to AA mismatch sites (Figure 4B) (14), which also contains an adjacent intercalation site (Figure 4C), and another of a binuclear complex, Δ,Δ-[μ-(11,11′-bidppz)(phen)_4_Ru_2_]^4+^, bound to d(CGTACG)_2_ (Figure 4D) (26). The question could be asked whether this mode of binding—insertion induced base flipping combined with π stacking by a purine—could be more generally applicable, and hence whether it may be relevant to observations made in solution.

When bound to DNA in solution, the luminescence lifetime and intensity of the ‘bright’ ^3^MLCT excited state has been reported to be inversely related to the number of hydrogen bonds that the dppz pyrazine nitrogens are able to form with solvent molecules. Thus, no luminescence is observed with the free compound in water while the strongest luminescence is observed when neither of the nitrogens is H-bonded to the solvent (27). The luminescence is therefore sensitive to changes in the orientation of the complex and the solvent environment around the compound (28). It has also been reported that the bound Δ enantiomer almost always luminesces more strongly than Λ, and that the strongest luminescence is observed for the Δ enantiomer (29) bound to A-T rich sequences. A reduction in luminescence is, broadly speaking, observed as AT (or CI, where I is inosine) content decreases (29–31). Whilst it has been reported that G can quench luminescence (29), the Λ enantiomer has a longer luminescence lifetime in poly(dG).poly(dC) than poly(dA).poly(dT). There must therefore be differences in the binding mode that the Δ adopts compared with the Λ.

**Figure 5.**
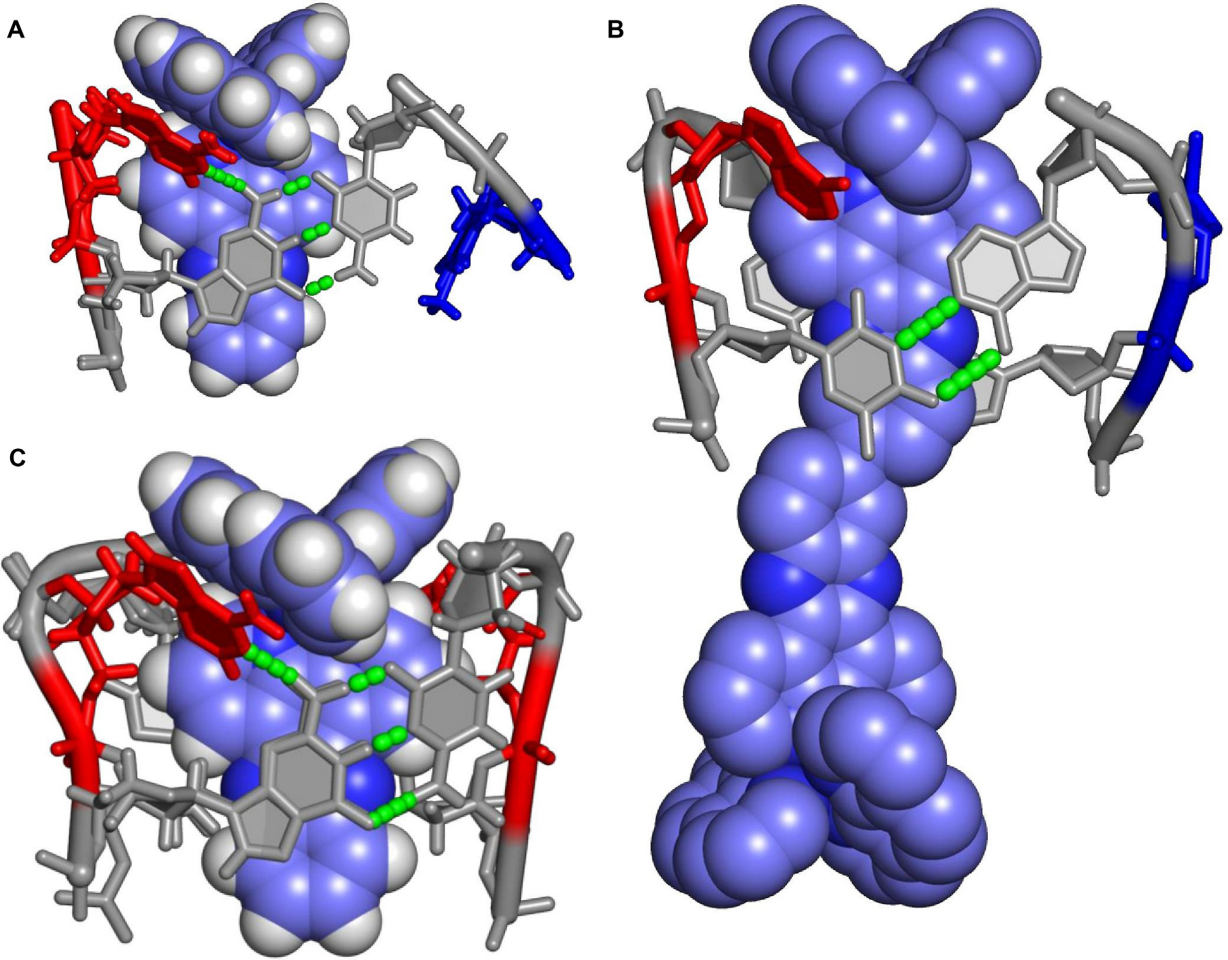
A comparison of the binding sites for Δ Ru-polypyridyls in DNA. (**A**) The binding site in the present work. Δ-[Ru(phen)_2_(dppz)]^2+^ binds into d(TCGGCGCCGA)_2_ at the terminal step, with T_1_ and A_10_ flipped out. A_10_ stacks onto an ancillary phen group. (**B**) A similar binding motif crystallised in the threading of Δ,Δ-[μ-(11,11′-bidppz)(phen)_4_Ru_2_]^4+^ at the TA/TA step of d(CGTACG)_2_. Both (B)T_3_ and (A)A_4_ are flipped out, with (A)A_4_ stacking on an ancillary phen group. (**C**) Δ-[Ru(bpy)_2_(dppz)]^2+^ crystallised at an AA mismatch site, with both adenine bases flipped out and stacked on the bpy ligand groups from the complex (PDB ID 4E1U). In all three structures, a hydrogen bond is formed between the flipped adenine and a G(NH_2_) on the 5′ side of the binding site, although this not adjacent to the threading cavity with Δ,Δ-[μ-(11,11′-bidppz)(phen)_4_Ru_2_]^4+^ (PDB ID 4GQJ). In this figure, adenine bases are drawn in red with thymine bases in blue. The atoms in the complex are coloured according to type with nitrogen in deep blue, carbon in light blue and hydrogen in white. Other DNA bases are drawn in grey. Hydrogen bonds between the bases are drawn as dashed lines in green.

In our structure we have half a binding site – the complex is interacting at the end of the duplex (formally it would be said to end-cap into the minor groove) and, where the T_1_–A_10_ base pair should be, is another complex. However, a structure reported by Song *et al*., shows Δ-[Ru(bpy)_2_(dppz)]^2+^ bound in two different environments in full binding sites (14) – one at AA mismatch sites and another at a 5′-AT-3′ step, flanked by the two flipped out adenines from the neighbouring mismatch steps. In both environments, the two bpy groups are flanked by π-stacking adenine bases in the minor groove, consistent with the binding motif observed in our structures. In a second structure, of a binuclear Δ,Δ complex bound to d(CGTACG)_2_ (26), the complex is threaded through a T-A base pair, causing both (B)T_3_ and (A)A_4_ to flip out, where (A) and (B) are the two chains which form the helix. (A)A_4_ then stacks onto one of the phen ligands, whilst T_3_ forms a Hoogsteen base pair with a symmetry related (A)A_4 ._ In structures 1 and 2, the A_10_ stacks with a phen group from the ligand and T_1_ also flips out but stacks onto the phen from a complex interacting with a neighbouring duplex. The angle between the long axis of the dppz group and the C_2_:G_9_ base pair is 62°, consistent with that observed for the both the mismatch site and the threaded structure. The overall binding motif in all three structures is remarkably similar (Figure 5). Flipping out of a well-matched base pair has also been reported in a partially refined structure, containing Δ-[Ru(bpy)_2_(dppz)]^2+^ bound at a well-matched step and intercalating from the minor groove (32). Base-flipping at a mismatch site, by a Δ enantiomer, was first observed in X-ray crystal structures containing Rh complexes (33,34).

**Figure 6.**
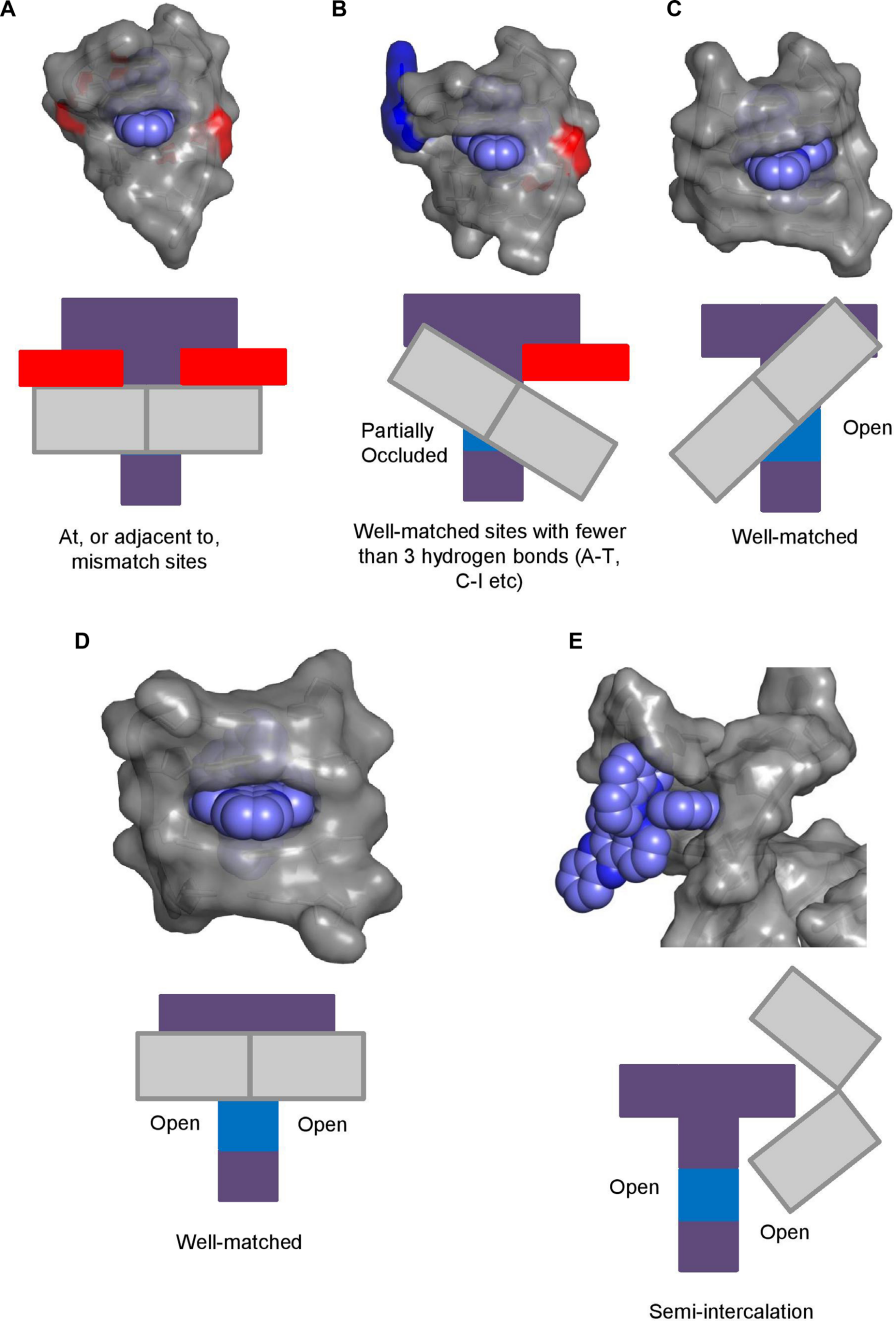
Five possible binding modes for Δ-[Ru(phen)_2_(dppz)]^2+^ to DNA. (**A**) The complex binds at, or adjacent to, a mismatch site. The flanking adenine (or purine) bases stack on the ancillary ligands, reducing intercalation depth and preventing the dppz nitrogen atoms from hydrogen bonding to solvent water molecules (14). (**B**) Insertion into well-matched sites with less than three H-bonds between the bases. The purine flips out and is able to π-stack onto an ancillary ligand. The pyrimidine also flips out but does not stack. This partially, but not completely, occludes one dppz nitrogen (26). (**C**) Canted (angled) intercalation into a well-matched base pair leaves one dppz nitrogen atom entirely exposed to solvent (8). (**D**) Model for intercalation by a Δ enantiomer at a 5′-AT/AT-3′ step. The model was generated by changing both the enantiomer and the base sequence (TA → AT) of the DNA step starting from PDB code 3U38(7). Symmetrical intercalation into this step should expose both dppz nitrogen atoms to the solvent. (**E**) Semi-intercalation by an ancillary ligand into the DNA duplex, exposing both phenazine nitrogen atoms to solvent. DNA is drawn in grey as a solvent-accessible surface with a radius of 1.5 Å. The carbon atoms of the complex are in purple with nitrogen atoms in blue. In the schematic diagram, the complex is drawn in purple with dppz nitrogen atoms as blue. DNA bases are represented by grey blocks with flanking adenine bases as red rectangles.

A consequence of binding at a mismatch, with two flanking purine bases stacking on the ancillary ligands, is that both pyrazine nitrogen atoms are not solvent accessible (Figure 6A). This, combined with the additional π-stacking, should provide a tight binding site with increased luminescence (14), as the dppz nitrogen atoms are not water accessible. However, if we consider the structure of Δ,Δ-[μ-(11,11′-bidppz)(phen)_4_Ru_2_]^4+^ bound into d(CGTACG)_2_, both an A and T are flipped out of the duplex (Figure 4D). The A stacks onto a phen but the T does not, which is consistent with the structures reported here and the partially refined structure by Song (32). This results in blocking of the phenazine N on the A side and partially occluding the N on the T side of the dppz (Figure 6B). Whilst one dppz nitrogen is accessible, it is buried deeper in the DNA base stack and therefore hydrogen bonding may be less favourable than if the site was completely open. We have also previously reported both enantiomers of [Ru(phen)_2_(dppz)]^2+^ bound to d(ATGCAT)_2_ (8), showing that the Δ enantiomer is bound at an angle, leaving one dppz nitrogen completely exposed to solvent. The complex would therefore be able to form a hydrogen bond with the solvent on one side only (Figure 6C).

In solution, interactions such as this would be expected to be observable by NMR. Only a small number of NMR studies, examining the interactions between a mononuclear ruthenium complex and DNA, have been reported. This could be due to the unfavourable association and dissociation times between the complex and DNA, causing peak broadening and significantly hampering the interpretation of such spectra, making such studies highly challenging.

The first study uses an innovative selective deuteration strategy to assign the intercalation site as from the major groove (35). Since then, a number of studies (36,37) have been reported and do not support the binding mode proposed in this present work because there are no significant chemical shifts for the protons in the ancillary ligands. These studies do show minor groove intercalation and, in some cases, give a molecular model of the binding site. However, in some of these the authors were unable to produce a model which explains all of the NOE distances observed, notably in two such reports where the minor component made up 15% of the observed binding (38,39). One explanation for this observation is that the complex could occupy a variety of binding sites but another is that the spectra are complicated by a second binding mode. A further study is different in that binding was assigned as a partial intercalation mode (40). However, significant shifts in the ^1^H spectrum were observed for the bpy groups in Δ-[Ru(bpy)_2_(HPIP)]^2+^. Significant shifts were also observed in the phenanthrolinic protons of the HPIP, with the bpy groups giving NOE crosspeaks with the H1′ sugar protons on G_4_, A_5_ and C_6_ in d(GTCGAC)_2,_ which could be consistent with the binding mode proposed here. More NMR studies would be necessary to confirm the presence, or absence, of insertion combined with base-flipping in solution, which could establish whether this binding mode is generally applicable.

We have previously reported a structure of Λ-[Ru(phen)_2_(dppz)]^2+^ bound to d(CCGGTACCGG)_2_ by symmetrical intercalation. If this structure is used as a model, the Λ enantiomer replaced with a Δ superimposed at the binding site, and the bases are reversed from 5′-TA-3′ to 5′-AT-3′ (consistent with the intercalation site in the mismatch structure by Song *et al*.), the Δ enantiomer fits almost perfectly. However, intercalation at this step would be deeper and therefore both dppz nitrogen atoms would be exposed to solvent (Figure 6D). Of course, semi-intercalation (Figure 6E) exposes both dppz nitrogen atoms to solvent in a completely unrestricted environment. We therefore suggest that the luminescence lifetime of the complexes in the binding sites presented here would be in the following order:

Mismatch (AA) **>** Well-matched, non CG site with base flipping **≥** canted intercalation **>** symmetrical intercalation **>** semi-intercalation.

Flipping out both bases, in an individual base pair, has not been reported for the Λ enantiomer and indeed it appears as if the geometry required to do this, and have the base π-stack in the same way, would be highly disfavoured. Whilst the flipping of a single base has been observed, in all cases a full base pair has been maintained even when the second base comes from a symmetry related duplex. One explanation for this is that inversion of chirality results in the placing of ancillary ligands into a non-optimal position for a flipped out base to stack, allowing a well-matched base pair to be maintained. As no binding mode has yet been reported which protects both dppz nitrogen atoms from solvent, we would expect the luminescence lifetime for the Λ enantiomer to be in the following order:

Canted intercalation **>** symmetrical intercalation **>** semi-intercalation.

We therefore propose that binding by the Δ enantiomer can induce base-flipping with stacking onto the ancillary ligand by purine base(s). This gives a binding mode with two different pyrazine N environments – one fully solvent inaccessible and the other partially accessible and this is unlikely to occur for the Λ enantiomer. This observation from crystallography could help to reinterpret measurements in solution and is likely to apply to a wide range of systems, including those containing photooxidising complexes where differences between the bound enantiomers have also been observed (41).

## Supplementary Material

SUPPLEMENTARY DATA

## ACCESSION NUMBERS

Coordinates and experimental data can be downloaded from www.wwpdb.org using PDB IDs 5JEU and 5JEV.
