# Supplementary material for: Delta chirality ruthenium ‘light-switch’ complexes can bind in the minor groove of DNA with five different binding modes
Source: Nucleic Acids Res. 2016 Sep 5;44(19):9472–82. doi: 10.1093/nar/gkw753 (PMC5100598; doi:10.1093/nar/gkw753)
Supplement: SUPPLEMENTARY DATA [file supp_44_19_9472__index.html]

Delta chirality ruthenium ‘light-switch’ complexes can bind in the minor groove of DNA with five different binding modes — Delta chirality ruthenium ‘light-switch’ complexes can bind in the minor groove of DNA with five different binding modes — Delta chirality ruthenium ‘light-switch’ complexes can bind in the minor groove of DNA with five different binding modes — SUPPLEMENTARY DATA 

# Delta chirality ruthenium ‘light-switch’ complexes can bind in the minor groove of DNA with five different binding modes

## SUPPLEMENTARY DATA

- SUPPLEMENTARY DATA
