# Supplementary material for: Delta chirality ruthenium ‘light-switch’ complexes can bind in the minor groove of DNA with five different binding modes
Source: Nucleic Acids Res. 2016 Sep 5;44(19):9472–82. doi: 10.1093/nar/gkw753 (PMC5100598; doi:10.1093/nar/gkw753)
Supplement: SUPPLEMENTARY DATA [file supp_gkw753_new-supp-SIv1.7.pdf]

Delta Chirality Ruthenium ‘Light-switch’ Complexes can Bind in the Minor Groove of DNA with Five Different Binding Modes

James P. Hall[1,2*], Páraic M. Keane[1], Hanna Beer[1], Katrin Buchner[1], Graeme Winter[2], Thomas L. Sorensen[2], David J. Cardin[1], John A. Brazier[3], Christine J. Cardin[1*]

[1] Department of Chemistry, University of Reading, Whiteknights, Reading, RG6 6AD, UK

[2] Diamond Light Source, Harwell Science and Innovation Campus, Fermi Avenue, Didcot, OX11 0DE, UK

[3] Department of Pharmacy, University of Reading, Whiteknights, Reading, RG6 6AD, UK

* To whom correspondence should be addressed. Tel:+441183788215; Email:c.j.cardin@reading.ac.uk
Correspondence may also be addressed to Dr James Hall. Tel: +441183786073; Email: james.hall@reading.ac.uk.

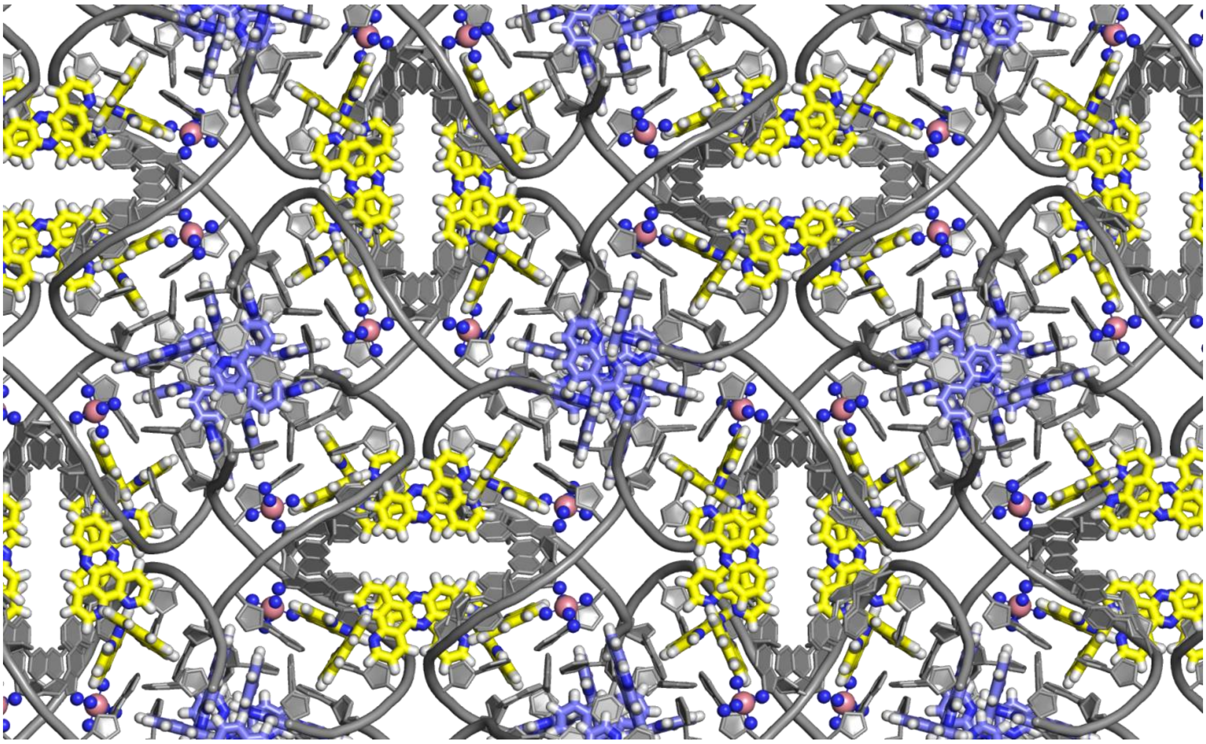

Figure S1 – A packing diagram, looking down the crystallographic 4_1 screw axis, of structure 2 (Δ-$[Ru(phen)_2(dppz)]^{2+}$ bound to $d(TCGGCGCCGA)_2$ with cobalt hexammine). End-capping complexes are drawn in with carbon atoms in blue with the carbons of semi-intercalating complexes in yellow and DNA as grey. Other atoms are coloured according to type with nitrogen in blue, hydrogen in white and cobalt in pink.

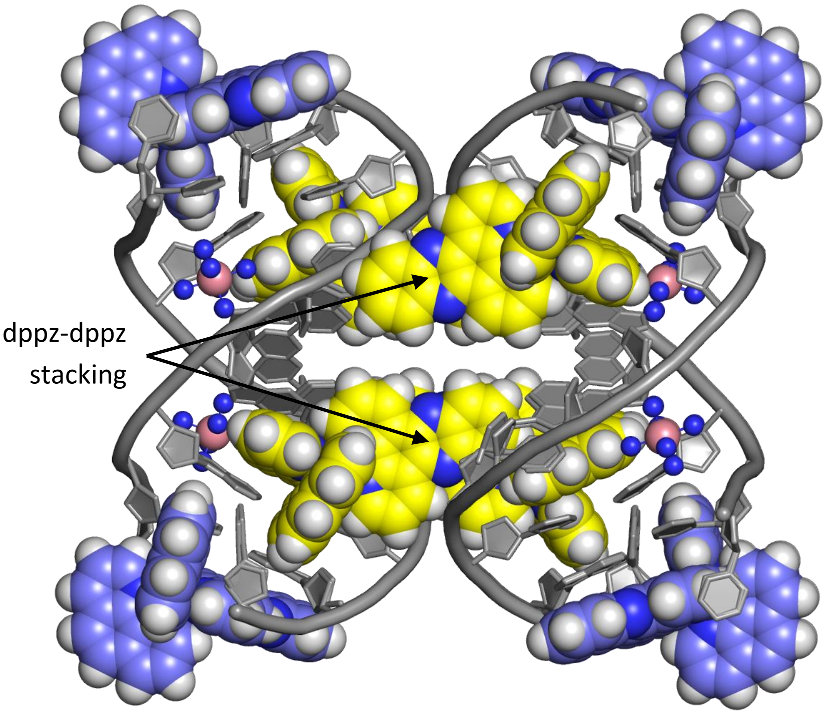

Figure S2 – Semi-intercalating dppz-dppz stacking between semi-intercalated complexes. End-capping complexes are drawn in with carbon atoms in purple with the carbons of semi-intercalating complexes in yellow and DNA as grey. Other atoms are coloured according to type with nitrogen in blue, hydrogen in white and cobalt in pink.

Table S1: Conformational analysis for structure 1 (with Ba^{2+})

****

Local base-pair parameters

bp	Shear	Stretch	Stagger	Buckle	Propeller	Opening
1 C-G	0.19	-0.18	0.32	-3.85	-10.34	-0.83
2 G-C	-0.20	-0.08	0.43	17.55	-6.03	-1.17
3 G-C	-0.29	-0.14	-0.16	-11.70	-1.22	-0.99
4 C-G	0.10	-0.08	0.23	-3.00	-5.42	-1.87
5 G-C	-0.10	-0.08	0.23	3.00	-5.42	-1.87
6 C-G	0.29	-0.14	-0.16	11.70	-1.22	-0.99
7 C-G	0.20	-0.08	0.43	-17.55	-6.03	-1.17
8 G-C	-0.19	-0.18	0.32	3.85	-10.34	-0.83
ave.	-0.00	-0.12	0.20	0.00	-5.76	-1.22
s.d.	0.22	0.04	0.24	11.57	3.46	0.42

****

Local base-pair step parameters

step	Shift	Slide	Rise	Tilt	Roll	Twist
1 CG/CG	0.07	0.42	2.89	4.82	1.33	30.42
2 GG/CC	-0.04	0.34	5.16	1.39	49.66	21.20
3 GC/GC	-0.88	-0.07	3.09	-3.74	2.94	33.03
4 CG/CG	0.00	0.26	3.09	0.00	6.09	32.11
5 GC/GC	0.88	-0.07	3.09	3.74	2.94	33.03
6 CC/GG	0.04	0.34	5.16	-1.39	49.66	21.20
7 CG/CG	-0.07	0.42	2.89	-4.82	1.33	30.42
ave.	-0.00	0.23	3.62	0.00	16.28	28.77
s.d.	0.51	0.21	1.05	3.61	22.86	5.28

****

Local base-pair helical parameters

step	X-disp	Y-disp	h-Rise	Incl.	Tip	h-Twist
1 CG/CG	0.55	0.71	2.88	2.52	-9.12	30.82
2 GG/CC	-5.05	0.20	2.41	68.30	-1.92	53.75
3 GC/GC	-0.58	0.94	3.15	5.14	6.53	33.36
4 CG/CG	-0.54	-0.00	3.08	10.89	-0.00	32.67
5 GC/GC	-0.58	-0.94	3.15	5.14	-6.53	33.36
6 CC/GG	-5.05	-0.20	2.41	68.30	1.92	53.75
7 CG/CG	0.55	-0.71	2.88	2.52	9.12	30.82
ave.	-1.53	0.00	2.85	23.26	-0.00	38.36
s.d.	2.46	0.69	0.32	30.90	6.57	10.57

****

Strand I

base	v0	v1	v2	v3	v4	tm	P	Puckering
1 C	-4.4	-13.9	25.9	-29.3	21.2	29.1	27.2	C3'-endo
2 G	0.8	-20.1	30.7	-30.8	19.2	32.1	17.1	C3'-endo
3 G	-19.2	32.3	-33.0	22.5	-2.2	34.1	165.2	C2'-endo
4 C	-21.6	30.8	-28.7	16.6	2.9	31.3	156.3	C2'-endo
5 G	-27.3	35.3	-30.1	15.0	7.4	35.0	149.3	C2'-endo
6 C	-30.1	4.4	20.9	-38.6	44.3	43.5	61.3	C4'-exo
7 C	-27.2	38.1	-34.7	19.8	4.3	38.2	155.0	C2'-endo
8 G	-36.1	45.6	-37.0	17.4	11.5	44.5	146.4	C2'-endo

Table S2: Conformational analysis for structure 2 (with cobalt hexammine)

Local base-pair parameters

bp	Shear	Stretch	Stagger	Buckle	Propeller	Opening
1 C-G	0.15	-0.14	0.21	-6.20	-8.09	-1.09
2 G-C	-0.15	-0.09	0.33	15.67	-2.83	-1.09
3 G-C	-0.13	-0.09	-0.03	-10.80	-7.14	-1.22
4 C-G	0.12	-0.16	0.01	2.46	-5.72	-0.77
5 G-C	-0.12	-0.16	0.01	-2.46	-5.72	-0.77
6 C-G	0.13	-0.09	-0.03	10.80	-7.14	-1.22
7 C-G	0.15	-0.09	0.33	-15.67	-2.83	-1.09
8 G-C	-0.15	-0.14	0.21	6.20	-8.09	-1.09
ave.	-0.00	-0.12	0.13	-0.00	-5.95	-1.04
s.d.	0.15	0.03	0.16	10.78	2.12	0.18

Local base-pair step parameters

step	Shift	Slide	Rise	Tilt	Roll	Twist
1 CG/CG	0.07	0.36	2.83	3.70	3.18	30.77
2 GG/CC	-0.52	0.11	5.27	0.70	48.60	23.60
3 GC/GC	0.14	-0.11	3.08	-2.66	2.49	28.93
4 CG/CG	-0.00	0.50	3.24	0.00	3.16	39.01
5 GC/GC	-0.14	-0.11	3.08	2.66	2.49	28.93
6 CC/GG	0.52	0.11	5.27	-0.70	48.60	23.60
7 CG/CG	-0.07	0.36	2.83	-3.70	3.18	30.77
ave.	-0.00	0.18	3.66	-0.00	15.96	29.37
s.d.	0.31	0.24	1.11	2.66	22.30	5.23

Local base-pair helical parameters

step	X-disp	Y-disp	h-Rise	Incl.	Tip	h-Twist
1 CG/CG	0.15	0.49	2.84	5.94	-6.92	31.15
2 GG/CC	-5.14	0.65	2.48	65.63	-0.95	53.72
3 GC/GC	-0.73	-0.83	3.03	4.97	5.29	29.16
4 CG/CG	0.38	0.00	3.27	4.72	-0.00	39.14
5 GC/GC	-0.73	0.83	3.03	4.97	-5.29	29.16
6 CC/GG	-5.14	-0.65	2.48	65.63	0.95	53.72
7 CG/CG	0.15	-0.49	2.84	5.94	6.92	31.15
ave.	-1.58	-0.00	2.85	22.54	0.00	38.17
s.d.	2.47	0.67	0.29	29.44	5.06	11.15

Strand I

base	v0	v1	v2	v3	v4	tm	P	Puckering
1 C	-2.4	-11.8	21.0	-22.9	15.9	23.1	24.4	C3'-endo
2 G	-1.7	-18.1	29.8	-31.6	21.3	32.1	21.7	C3'-endo
3 G	-12.6	29.6	-34.7	28.0	-9.9	34.7	177.7	C2'-endo
4 C	-38.2	46.6	-36.9	16.2	13.5	45.6	144.2	C2'-endo
5 G	-22.4	0.3	20.2	-33.7	35.4	36.0	55.8	C4'-exo
6 C	-11.5	-11.0	27.7	-35.2	29.8	34.9	37.5	C4'-exo
7 C	-27.6	37.5	-33.6	18.3	5.5	37.6	153.2	C2'-endo
8 G	-33.0	45.6	-39.9	22.0	6.6	44.9	152.8	C2'-endo
